# Differential Effects of the Inactivation of Anterior and Posterior Orbitofrontal Cortex on Affective Responses to Proximal and Distal Threat, and Reward Anticipation in the Common Marmoset

**DOI:** 10.1093/cercor/bhab240

**Published:** 2021-09-08

**Authors:** Zuzanna M Stawicka, Roohollah Massoudi, Lydia Oikonomidis, Lauren McIver, Kevin Mulvihill, Shaun K L Quah, Gemma J Cockcroft, Hannah F Clarke, Nicole K Horst, Christian M Wood, Angela C Roberts

**Affiliations:** Department of Physiology, Development and Neuroscience, University of Cambridge, Cambridge CB2 3DY, UK; Behavioral and Clinical Neuroscience Institute, University of Cambridge, Cambridge CB2 3EB, UK; Department of Physiology, Development and Neuroscience, University of Cambridge, Cambridge CB2 3DY, UK; Behavioral and Clinical Neuroscience Institute, University of Cambridge, Cambridge CB2 3EB, UK; Department of Physiology, Development and Neuroscience, University of Cambridge, Cambridge CB2 3DY, UK; Behavioral and Clinical Neuroscience Institute, University of Cambridge, Cambridge CB2 3EB, UK; Department of Physiology, Development and Neuroscience, University of Cambridge, Cambridge CB2 3DY, UK; Behavioral and Clinical Neuroscience Institute, University of Cambridge, Cambridge CB2 3EB, UK; Department of Physiology, Development and Neuroscience, University of Cambridge, Cambridge CB2 3DY, UK; Department of Physiology, Development and Neuroscience, University of Cambridge, Cambridge CB2 3DY, UK; Behavioral and Clinical Neuroscience Institute, University of Cambridge, Cambridge CB2 3EB, UK; Department of Physiology, Development and Neuroscience, University of Cambridge, Cambridge CB2 3DY, UK; Behavioral and Clinical Neuroscience Institute, University of Cambridge, Cambridge CB2 3EB, UK; Now at Cambridge Centre for Teaching and Learning, University of Cambridge, Cambridge CB2 3PT, UK; Now at Postdoc Academy, University of Cambridge, Cambridge CB3 1AS, UK; Department of Physiology, Development and Neuroscience, University of Cambridge, Cambridge CB2 3DY, UK; Behavioral and Clinical Neuroscience Institute, University of Cambridge, Cambridge CB2 3EB, UK; Department of Physiology, Development and Neuroscience, University of Cambridge, Cambridge CB2 3DY, UK; Behavioral and Clinical Neuroscience Institute, University of Cambridge, Cambridge CB2 3EB, UK

**Keywords:** anxiety, reward, orbitofrontal, Pavlovian, threat

## Abstract

Structural and functional abnormalities of the orbitofrontal cortex (OFC) have been implicated in affective disorders that manifest anxiety-related symptoms. However, research into the functions of primate OFC has predominantly focused on reward-oriented rather than threat-oriented responses. To redress this imbalance, the present study performed a comprehensive analysis of the independent role of 2 distinct subregions of the central OFC (anterior area 11; aOFC and posterior area 13; pOFC) in the processing of distal and proximal threat. Temporary inactivation of both aOFC and pOFC heightened responses to distal threat in the form of an unknown human, but not to proximal threat assessed in a discriminative Pavlovian conditioning task. Inactivation of the aOFC, however, did unexpectedly blunt conditioned threat responses, although the effect was not valence-specific, as conditioned appetitive responses were similarly blunted and appeared restricted to a discriminative version of the task (when both CS^−^ and CS^+^ are present within a session). Inactivation of the pOFC did not affect conditioned responses to either proximal threat or reward and basal cardiovascular activity was unaffected by manipulations of activity in either subregion. The results highlight the contribution of aOFC and pOFC to regulation of responses to more distal uncertain but not proximal, certain threat and reveal their opposing contribution to that of the immediately adjacent medial OFC, area 14.

## Introduction

The orbitofrontal cortex (OFC) has been implicated in a number of affective disorders, including depression (Drevets 2007; Cheng et al. 2016) and anxiety disorders (Milad and Rauch 2007). While this implies that the OFC may have an important role in the regulation of affective responses, its specific contributions remain unclear. There have been numerous psychological theories proposed as to the functions of the OFC (Roesch et al. 2007; Wilson et al. 2014; Padoa-Schioppa and Conen 2017; Murray and Rudebeck 2018). Most of these theories, however, have been developed based on findings from experiments investigating reward-directed behaviors, such as those testing reward valuation and economic decision-making (Gallagher et al. 1999; Schultz and Tremblay 1999; O’doherty et al. 2000; Schoenbaum et al. 2003; Izquierdo et al. 2004; Padoa-Schioppa and Assad 2006; West et al. 2011; Groman et al. 2019) in humans, monkeys, and rats. Comparatively less work has focused on the processing of threatening information and threat-elicited responses and behaviors (Agustín-Pavón et al. 2012; Clarke et al. 2015; Pujara et al. 2019). This is despite the fact that human neuroimaging literature links not only the OFC with affective disorders but also trait anxiety (Roppongi et al. 2010; Indovina et al. 2011), a risk factor for developing affective disorders and a trait that affects decision-making under threat (Fung et al. 2019).

Revealing the contribution of the OFC as distinct from other regions of the prefrontal and cingulate cortex to such traits and disorders is an important step forward in determining the neurobiological basis underlying the marked heterogeneity in the etiology and also treatment of affective disorders. Heterogeneity is considered one of the major causes of the current impasse in effective treatments for anxiety and depression and underlies the drive for individualized treatment strategies (Insel and Cuthbert 2009; Blom et al. 2014). A conceptual platform upon which such heterogeneity may be identified is that crystallized by Fanselow and Lester (1988) and recently elaborated by Mobbs et al. (2020). By defining threat in terms of its proximity in time, space, and probability, the different behavioral and cognitive strategies adopted and hence the different neurobiological circuits engaged in response to threat can be understood. For example, based on human neuroimaging studies, it has been proposed that while mid-cingulate cortex and subcortical regions are engaged in response to proximal threat, when the threat is certain and the emphasis is on relatively rapid responses, processing in prefrontal regions is engaged primarily in response to more distal threat, including when the threat level may be uncertain and there is time and opportunity to employ a range of higher-order executive functions to contribute to the decision making process (Qi et al. 2018). Thus, it is important for translational studies to compare responsivity to these different types of uncertain and distal threat and more certain, proximal threat when studying the causal involvement of distinct prefrontal regions.

An additional consideration when studying the OFC is its distinct subregions based on dissociable cytoarchitectonic features and connectivity patterns. Such subregions exist in both primates and rodents, but while these are generally comparable across human and nonhuman primates, their analogous/homologous counterparts in rodents are as yet unclear. In primates, the subdivision of the orbital surface of the macaque monkey by Walker (1940) and later reproduced and refined by Carmichael and Price (1994), Petrides (1994), and Barbas and Pandya (1989), differentiates between medial area 14 (gyrus rectus), also considered part of the ventromedial prefrontal cortex (vmPFC; for review of anatomical nomenclature, see Roberts and Clarke 2019), frontal pole area 10, central anterior area 11 (anterior portion of the orbital gyrus, from the orbital to the frontomarginal sulcus), central posterior area 13 (posterior portion of the orbital gyrus), and lateral area 12 (also denoted as 47/12; Petrides 1994). Importantly, the same anatomical subdivisions have been recognized in the human (Öngür et al. 2003; Mackey and Petrides 2010).

Here, we are focusing on central OFC, specifically comparing the anterior and posterior divisions, area 11 and 13, respectively, in a new world primate, the common marmoset. Previously, permanent excitotoxic lesions of the anterior division in marmoset heightened responsivity to more uncertain, distal threat as measured by the human intruder test and, while not affecting the expression of Pavlovian conditioned proximal threat responses, did cause those responses to become inflexible to changes in stimulus-outcome contingencies (Agustín-Pavón et al. 2012). Heightened threat reactivity was also recently reported following combined excitotoxic lesions of areas 11/13 in old world macaque monkeys (Pujara et al. 2019). Based on the reward valuation literature, however, these 2 regions can be dissociated with a specific role postulated for the more posterior region (mostly area 13) in real-time value updating following satiation of an unconditioned rewarding stimulus, whereas the more anterior region (mostly area 11) has been implicated instead in using the updated value information to guide subsequent choice between 2 food options signaled by distinct stimuli (Murray et al. 2015). However, the potentially distinct contributions of areas 11 and 13 to the processing of, and reactivity to, threat have not been investigated.

In the current study, the anterior (a)OFC and posterior (p)OFC of the marmoset were therefore the subject of a systematic comparative analysis of their contribution to reactivity to uncertain and thus more distal threat and certain, proximal threat. It was hypothesized that the central OFC would contribute to distal but not proximal threat processing based on the findings from human neuroimaging studies (Mobbs et al. 2020). Marmosets were implanted with chronic indwelling cannulae into either the aOFC (primarily area 11) or pOFC (primarily area 13) or both, allowing for the temporary manipulation of one or other region. The human intruder test, an experimental paradigm historically used in primates, was used as a test of reactivity to a more distal threat in the form of an unfamiliar human, considered a post encounter, uncertain threat. The effect of aOFC and pOFC inactivation on this test was compared with that seen on conditioned cardiovascular and behavioral responses to certain, proximal threat, as measured in a test of discriminative Pavlovian conditioning to an imminent aversive stimulus, considered a circa-strike threat. The effects of manipulations were also analyzed on basal cardiovascular activity in a neutral setting, as a control for the threat-elicited cardiovascular measurements, but also because cardiac dysfunction has been linked to affective disorders (Carney et al. 2001; Nemeroff and Goldschmidt-Clermont 2012; Batelaan et al. 2016). To directly compare the role of aOFC and pOFC in threat and reward processing, the effects of inactivation of aOFC and pOFC were also investigated on discriminative Pavlovian conditioning to reward using an almost identical paradigm to that used for threat conditioning except that the CS was associated with reward rather than punishment. Although permanent excitotoxic lesions of the aOFC are without effect on the expression of Pavlovian conditioned responses (Roberts et al. 2007; Reekie et al. 2008), it was important to determine whether similar or different effects were seen following temporary inactivation.

## Materials and Methods

### Subjects and Housing

Subjects were 16 common marmosets (9 male, 7 female; *Callithrix jacchus*), bred on site at the University of Cambridge Marmoset Breeding Colony. Thirteen marmosets were experimentally naïve, while the remaining 3 had taken part in experiments not included in this publication (Table 1). All marmosets were housed in male–female pairs (males were vasectomized) in stainless steel cages containing environmental enrichment, such as ladders, ropes, and platforms. The temperature in the holding rooms was maintained at 22 ± 1 °C, with humidity at 50 ± 1%. The lights were operated on a 12-h light–dark schedule, gradually brightening between 7 and 7:30 am and dimming between 7 and 7:30 pm. The marmosets were provided with water ad libitum and were given a morning feed of rusk with fruit (banana, grape, pear, or apple), as well as an afternoon feed of sandwich (bread, Complan, Mazuri powder, egg) and fruit. Marmoset welfare was monitored continuously by researchers as well as technical staff. All research was conducted in accordance with the UK Animals (Scientific Procedures) Act 1968 and the University of Cambridge Animal Welfare and Ethical Review Board.

**Table 1:** Full list of subjects taking part in the studies

Subject	Symbol	Cannulae	Human intruder test	Basal cardiovascular	Threat conditioning	Appetitive conditioning
S1	☆	aOFC	✓			
S2	⊙		✓			
S3	⊡		✓			
S4	⊞		✓			
S5	⊗			✓^1^		✓^2^
S6	⊠			✓^1^		✓^2^
S7	◇	Combined aOFC		✓^1^		✓^2^
S8	△	and pOFC	✓^3^	✓^1^	✓^2^	✓^3^
S9	▽			✓^1^	✓^2^	
S10	▢		✓^3^	✓^1^	✓^2^	✓^3^
S11	〇		✓^3^	✓^1^	✓^2^	✓^3^
S12	⎔			✓†		
S13	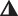	pOFC	✓			
S14	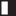		✓			
S15	◑		✓			
S16	x		✓			

Note: The table indicates which animals had cannulae implants into aOFC, pOFC, or both and which experimental procedures the animals took part in. The superscript numbers indicate the order the experiments were carried out in for subjects S5–S11. Infusions for the human intruder test were carried out while animals were also being trained on the Pavlovian appetitive task. However, they received no Pavlovian training on days when they received a human intruder test. Symbols for each subject are used throughout the paper. Subjects S1–S4 took part in an approach-avoidance decision-making test (such as that described in Clarke et al. 2015), data from which is not included in this paper. Infusions on the Human Intruder test were performed before any subsequent infusions on the decision-making task. Subjects S5–S7 had failed to learn the decision-making task and were subsequently cannulated and tested on basal cardiovascular and appetitive conditioning only. Subjects S8–S16 were naïve. Subject S9 did not go on to receive human intruder and the appetitive discrimination because of delays due to COVID restrictions in the lab and S12 only contributed to basal cardiovascular measurements and did not progress to further studies due to welfare reasons.

### Surgical Procedures

All marmosets underwent a procedure implanting chronic cannulae into the anterior (area 11) or posterior (area 13) OFC. Eight of the 16 marmosets also underwent a surgery to implant a telemetry device for the transmission and recording of real-time cardiovascular activity.

#### For All Surgical Procedures

Marmosets were premedicated with ketamine hydrochloride (0.1 mL of 100 mg/mL solution i.m., Vetalar, Amersham Biosciences and Upjohn) and received the nonsteroidal anti-inflammatory carprofen (0.03 mL of 50 mg/mL solution s.c., Carprieve, Norbrook). Anesthesia was then induced through intubation and delivery of 2.0–2.5% isoflurane gas in 0.3 L/min of oxygen. Respiration, heart rate (HR), oxygen saturation, and carbon dioxide blood levels were monitored throughout all surgeries using a pulse oximeter capnograph (Microcap Handheld Capnograph, Oridion Capnography). Body temperature was monitored using a rectal temperature probe (TES-1319 K-type digital thermometer) and maintained using a heat mat. Following surgery, marmosets were monitored to ensure they were capable of maintaining oxygen saturation above 95% and placed in a heated incubator for recovery. Having fully recovered from anesthesia, they were returned to the home cage. Postoperative analgesia was administered using meloxicam (0.1 mL of 1.5 mg/mL suspension orally, Metacam, Boehringer Ingelheim) on the 3 days following surgery.

#### For the Implantation of a Telemetry Device

Surgery was carried out as previously described (Alexander et al. 2019). Following the sedation and induction of anesthesia (as above), the marmoset was placed on their back and an incision was made down the midline of the abdomen. The descending aorta was visualized. Blood flow was occluded by an assisting surgeon for a period of no longer than 3 min, while the lead surgeon made a small incision in the blood vessel and inserted the end of a telemetry probe (HD-S10 transmitter, Data Sciences International) into the aorta. The vessel and probe were sealed using a cellulose patch and tissue adhesive (Vetbond, 3M) and the transmitter was sutured in the abdomen. Perioperative treatment for this surgery was supplemented with antibiotics. Five of the 8 marmosets received 1 pre- and 6 postoperative doses of clavulanate-potentiated amoxicillin (0.25 mL of 50 mg/mL suspension orally, Synulox, Zoetis). The remaining 3 marmosets received a single oral dose of enrofloxacin on the day before (0.2 mL of 2.5% solution, Baytril, Bayer) and 1 dose immediately prior to surgery (0.2 mL of 25 mg/mL injectable solution, s.c., Baytril, Bayer; antibiotic regime was refined to reduce postoperative drug administration and the resulting disturbance to the marmosets).

#### For the Implantation of Bilateral Chronic Cannulae into aOFC and pOFC

Surgery was carried out as previously described (Clarke et al. 2015). Following the sedation and induction of anesthesia (as above), marmosets were placed in a stereotaxic frame designed for marmosets (David Kopf). Holes were drilled in the skull at the required coordinates. For marmosets individually cannulated in the aOFC, the anteroposterior coordinate (AP) was +17.00 from the interaural line, with lateromedial coordinate (LM) at ±3.00 from the sagittal sinus. For marmosets individually cannulated in the pOFC, the AP was +15.00 and LM was ±4.00. For marmosets cannulated in both the aOFC and pOFC, the AP coordinates remained the same, with the LM modified to ±3.05 for the aOFC and ±3.95 for the pOFC to accommodate for the 1.5 mm c/c distance (coordinates were modified in situ as previously described; Dias et al. 1997). Stainless steel cannulae (Plastics One) were then inserted into the brain. For subjects S1–S6, the cannulae were single 6 mm guides implanted bilaterally into the aOFC/area 11. For subjects S13-S16, the cannulae were single 8 mm guides implanted bilaterally into the pOFC/area 13. For the remaining subjects, S7-S12, the cannulae were double guides (1.5 mm c/c), with one of the stems 5 mm and the other 7 mm in length, targeting both area 11 and area 13 (see Table 1 for full list of subjects and placements). Cannulae were implanted with the aim of reaching to 2 mm above the base of the skull. The implant was then fixed into place using skull screws (Plastics One), SuperBond C&B adhesive resin, and dental acrylic (Paladur, Kulzer). The skin was sutured around the implant, and the cannulae were occluded with dummy cannulae (Plastics One) and covered with caps.

### Intracerebral Drug Infusions

Drug infusions were conducted in sterile conditions, as previously described (Clarke et al. 2015). All marmosets were gradually habituated to the entire procedure. For the drug infusions, the marmosets were gently restrained and brought into the procedure room. The protective caps and dummies were removed and the implant was cleaned with 70% isopropyl alcohol wipes (Alcotip, Universal). Injectors (Plastics One) connected to a 10 μL Hamilton syringe using PTFE tubing were carefully inserted into the guide cannulae. The PTFE tubing was prefilled with saline solution, as well as the substance to be infused, separated by a small air bubble. For purposes of inactivation, 0.5 μL of muscimol (0.1 mM, GABA_A_ receptor agonist, Sigma-Aldrich) and baclofen (1.0 mM, GABA_B_ receptor agonist, Sigma-Aldrich) in saline were infused bilaterally into the brain over 2 min (Mus-Bac). The control was a 2-min infusion of 0.5 μL of sterile saline. The injectors were kept in place for a further 1 min, to ensure diffusion of the drugs into the surrounding tissue. The injectors were then removed, and dummies and caps replaced. Marmosets were returned to the home cage for a period of 25 min, before they were retrieved or prepared for behavioral testing in the home cage. Stimulation of activity (overactivation) of the aOFC and pOFC was also examined in an experiment looking at basal cardiovascular activity, as a comparison to effects of overactivation of area 25 (Alexander et al. 2020), as well as early studies of area 11 and area 13 electrical stimulation in macaques (Hall et al. 1977). Overactivation was achieved with the bilateral infusion of the mGlu2/3 receptor antagonist LY341495 (1 ng/μL; Tocris Bioscience) combined with the GABA_B_ receptor antagonist, CGP52432 (1 ng/μL; Tocris Bioscience), referred to as CGP-LY. 1.0 μL of CGP-LY was infused over a period of 2 min, with a 15-min pretreatment time (as previously described; Wallis et al. 2019).

The results for each animal were derived from single control and treatment infusions. The order of behavioral tests on which infusions were performed is indicated in Table 1. The control and treatment infusions took place at least 1 week but no more than 4 weeks apart. There were no more than 24 infusions per site per animal over the course of the experimental life of the marmosets to prevent any damage to the target brain areas.

### Behavioral Testing

#### Behavior Testing Apparatus

For 3 of the 4 experiments, marmosets were tested in a specially designed sound-attenuated testing apparatus in a separate room (the remaining experiment took place in the home cage). Marmosets were initially trained to enter a Perspex carry box in exchange for a small amount of marshmallow reward. The box was brought to the testing room and slotted into a chamber within the testing apparatus. The chamber was fitted with a houselight (LED light strip, 3W), 3 cameras attached to the top of the chamber, and a speaker (Behringer) and microphone concealed behind a panel. A telemetry receiver for the relaying of cardiovascular data was located under the floor of the chamber (Physiotel, Data Sciences International). All data from the receiver were recorded and analyzed using Spike 2 software (Cambridge Electronic Design). Video footage obtained using the cameras in the chamber was recorded using Power Director software (Cyberlink).

#### Post-encounter Distal Threat in the Form of an Unknown Human Intruder

This experiment was conducted in the home cage, as previously described (Agustín-Pavón et al. 2012). Briefly, the marmoset was separated from their cage mate in the upper right quadrant of the cage. The behavior of the marmoset was recorded using a camera (GoPro Hero 5) standing at a short distance from the front of the cage, and vocalizations were recorded using a microphone (Sennheiser MKE 400). There was an initial 8-min separated phase, where all experimenters left the room and the marmoset was left alone in the divided section. The intruder (a researcher concealed with an unfamiliar realistic rubber mask of a human face [Masks Direct] and wearing a familiar gown and scrubs) entered the room and stood at a distance of 40 cm from the cage. The intruder made eye contact with the marmoset for 2 min, before leaving the room again. The behavior of the marmoset was then recorded for a further 5 min. A maximum of one human intruder test was carried out every 2 weeks to prevent habituation. The different masks used were counterbalanced between marmosets. The order of infusions (saline vs. Mus-Bac) remained the same, with the control session always preceding the inactivation. This order was chosen because pilot data suggested a heightening of anxiety with inactivation of aOFC and pOFC, an effect which runs counter to the direction of any change caused by habituation with repeated human intruder tests. If inactivation sessions had preceded control sessions, then it could not be determined whether the lower EFA scores following control infusions compared with inactivation infusions were due to habituation or an inactivation-induced increase in anxiety-like behavior. Regardless, as will be seen in the results, there was no evidence of habituation between control sessions consistent with our past research showing consistent responses to the human intruder with repeated sessions (Mikheenko et al. 2015).

Marmosets are known to display a range of different behaviors in response to a human intruder (Agustín-Pavón et al. 2012; Quah et al. 2020). Video recordings were analyzed in JWatcher software to quantify the proportion of time spent at the front and back of the cage, the average height in the cage, locomotion, and head and body bobs (rapid side-to-side upper body movements). The audio footage was analyzed using Syrinx software to identify the tsik mobbing call, as well as vigilance calls such as tsik-egg, tse-egg, and egg calls. This information was then used to calculate a composite score of anxiety-like behavior. This score is based on an exploratory factor analysis (EFA), which was conducted on 171 marmosets in the colony, and identified a single latent factor explaining 39.7% of the variance (Quah et al. 2020). The pattern of behaviors and their relative contributions to this score was consistent with anxiety-like behaviors commonly observed in marmosets.

#### Basal Cardiovascular Activity in an Affectively Neutral Setting

Marmosets were initially habituated to the sound-attenuated testing apparatus (described above). Marmosets were tested once a day, 5 days a week (Monday to Friday). They were placed inside the testing chamber for 5 min, with the duration of time spent inside gradually increased across each subsequent daily session until the maximum time of 20 min was reached. The house light was kept on and no sounds were played, in order to create an affectively neutral setting. Behavior was monitored to ensure that marmosets were not in distress, and the cardiovascular data recorded for each session. Once HR and systolic blood pressure (sysBP) showed stability across at least 3 consecutive sessions, intracerebral drug infusions began. Only 1 drug infusion per week was carried out and was counterbalanced across marmosets with some animals receiving saline, Mus-Bac, and CGP-LY infusions into the aOFC before the pOFC or vice versa. Following the infusion and pretreatment time, marmosets were placed in the testing apparatus for 20 min. HR and sysBP across the entire session were analyzed. Additionally, heart rate variability (HRV) was calculated using both the root mean square of successive differences (RMSSD) and Toichi’s cardiosympathetic and cardiovagal indices (CSI and CVI, respectively). The values were extracted from R-R interval data using Kubios HRV software.

#### Discriminative Conditioning to a Proximal Threat

The Pavlovian conditioning paradigm was introduced in the same apparatus as that used to measure cardiovascular activity in an affectively neutral setting (Alexander et al. 2020). Marmosets were tested once a day, 5 days a week (Monday to Friday). Initially, marmosets were trained to discriminate between 2 neutral tones (conditioned stimuli, or CS, “clicks” and “tone,” 20 s, 75 dB), which predicted 2 different outcomes or unconditioned stimuli (US): the US^+^ (30 s of darkness with 10 s of mildly aversive white noise at 80 dB) and the US^−^ (0.5-s 2 kHz beep at 75 dB). The 10-s period of white noise was pseudo-randomized to occur either within the first, middle, or last 10 s of the 30-s period of darkness. The 2 neutral tones were initially tested in a single preconditioning session to assess the subjects’ intrinsic responses to the “to-be conditioned” tones. Whichever tone produced the higher cardiovascular arousal was selected to be the CS^−^ (tone associated with the US^−^) and the remaining tone, the CS^+^ (tone associated with the US^+^), to ensure that any cardiovascular arousal seen during conditioning reflected conditioning per se. The discrimination training consisted of test sessions 7–12 min in length, with different combinations of CS presentations or trials. Each session contained 2–4 trials, with a maximum of 1 CS^+^-US^+^ presentation per session and a maximum of 2 CS^+^-US^+^ per week. This gave a number of interspersed session types, including 2 CS^−^, 3 CS^−^, 2 CS^−^ with 1 CS^+^, or 3 CS^−^ with 1 CS^+^ presentations. The CS^+^ was never presented as the first trial of the session.

Conditioning was assessed by looking at behavioral and cardiovascular arousal. Behavioral arousal was assessed by recording the duration of vigilant scanning, a behavior associated with rapid side to side head movements and a raised, tense body posture of the marmoset, as previously described (Mikheenko et al. 2010). The cardiovascular arousal was assessed using the HR measure and not blood pressure, because the conditioned increase in blood pressure was less consistent across marmosets, as previously described (Mikheenko et al. 2010). The reasons for greater consistency of HR over blood pressure on the aversive conditioning task in marmosets are unclear. However, it is worth noting that HR is the cardiovascular measure of autonomic arousal consistently used in human studies of conditioned responses to threat (Lonsdorf et al. 2017).

The cardiovascular and behavioral arousal was quantified by looking at the 20-s CS period relative to the immediately preceding 20 s of baseline (CS-BL, also referred to as CS-directed responses). Successful conditioning was determined on the basis of consistent increases in CS^+^-directed, but not CS^−^-directed, cardiovascular and behavioral arousal over at least 3 sessions. Responses to the threat itself, the US, were also quantified by comparing the US to the preceding CS (US-CS, or US-directed).

Once successful conditioning was established, intracerebral infusions began. There was a maximum of 1 infusion per week. The order of infusions was counterbalanced between marmosets with some animals receiving saline and Mus-Bac infusions into the aOFC before the pOFC or vice versa. All infusions were followed by a specific session type, which consisted of an initial CS^−^-US^−^ trial, followed by a CS^+^-US^+^ trial, and a final CS^−^-US^−^ trial.

#### Discriminative Conditioning to Reward

The Pavlovian conditioning paradigm was introduced in a distinct apparatus from the one used for discriminative conditioning to threat. The testing apparatus was fitted with a special slot for the transparent carry box. From the carry box, the marmosets had access to 2 food boxes on either side of the chamber. The food boxes were gated with revolving doors, which could be set to closed, closed but transparent (inside of food box visible), or open (marmoset can reach into the food box) positions. Marmosets were tested once a day, 5 days a week (Monday to Friday). Marmosets were initially trained to retrieve reward from the food box. They were then trained to discriminate between 2 neutral tones (conditioned stimuli, or CS, “dreamharp” and “bubbles,” 20 s, 75 dB), which predicted 2 different outcomes or US: the US^+^ (120 s of an open food box full of mini marshmallows) and the US^−^ (an empty food box becoming visible for 120 s). The 2 neutral tones were initially tested in a single preconditioning session to assess the subjects’ intrinsic responses to the “to-be conditioned” tones, as described above for the threat conditioning procedure. The discrimination training consisted of test sessions 4–7 min in length, with different combinations of CS presentations or trials. Each session contained only 1 or 2 trials, with a maximum of 1 CS^+^-US^+^ presentation per session and a maximum of 5 CS^+^-US^+^ every 2 weeks. This gave a number of interspersed session types, including a single CS^−^, 2 CS^−^, a single CS^+^, or a CS^−^ followed by a CS^+^ presentation. The CS^+^ was never presented before a CS^−^, as the test session was terminated immediately after the CS^+^-US^+^. This was due to the fact that the food box door could not be closed again as these risked marmosets trapping their hands in the mechanism while reaching inside.

Conditioning was assessed by looking at behavioral and cardiovascular arousal. Behavioral arousal was assessed by a count of head jerks, a rapid side to side head flicking motion, as previously described in marmosets during appetitive conditioning (Braesicke et al. 2005). The cardiovascular arousal was assessed by recording of sysBP, as this measure was shown to provide the most consistent evidence of conditioned cardiovascular arousal to reward (Braesicke et al. 2005). Why appetitive stimuli appear to induce a more consistent effect on blood pressure rather than HR in marmosets is unclear. However, research in rodents has previously demonstrated a greater sensitivity of blood pressure, rather than HR, to feeding behaviors compared with other behaviors (LeDoux et al. 1982). The differences may also be mediated by differential activation of the sympathetic and parasympathetic nervous system, as the sympathetic nervous system has a direct influence on HR as well as sysBP, while the parasympathetic nervous system only has a direct impact on HR (Gordan et al. 2015). As before, the responses were quantified by looking at the 20-s CS period relative to the immediately preceding 20 s of baseline (CS-BL or CS-directed responses). Successful conditioning was determined on the basis of consistent increases in CS^+^-directed, but not CS^−^-directed, cardiovascular and behavioral arousal over at least 3 sessions. Responses to the reward itself, the US, were also quantified by comparing the US to the preceding CS (US-CS, or US-directed). Additionally, consumption of reward was monitored by weighing the amount of marshmallow consumed by the marmoset in the session.

Once successful conditioning was established, intracerebral infusions began. There was a maximum of 1 infusion per week. The order of infusions was counterbalanced between marmosets with some animals receiving saline and Mus-Bac infusions into the aOFC before the pOFC or vice versa. All infusions were followed by a session which consisted of a CS^−^-US^−^ trial followed by the CS^+^-US^+^ trial, after which the session was immediately terminated.

### Histological Analysis

**Figure 1:**
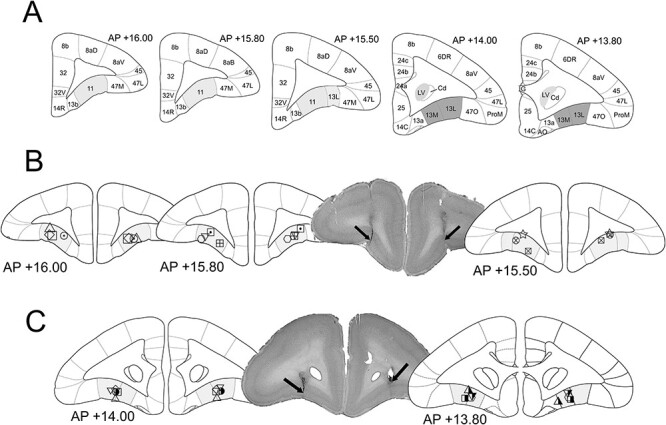
Confirmed cannula placements. The diagram presents schematized coronal sections of the marmoset brain, as seen in the marmoset brain atlas (Paxinos et al. 2012). (*A*) Unilateral coronal sections of the marmoset brains with the anatomical subdivisions. Targets for aOFC cannulation (light grey) and pOFC (dark grey) are highlighted (LV, lateral ventricle; Cd, caudate; ProM, preisocortical motor region; AO, anterior olfactory nucleus). Cannula placements are presented for each subject with a unique symbol within the aOFC (*B*) and the pOFC (*C*). The list of subjects represented by each symbol is presented in Table 1. The figure also includes 2 sample micrographs showing the cannula placements, for both area 11 and area 13, in tissue stained with cresyl violet (subject ▢).

At the end of the study, marmosets were sedated with ketamine hydrochloride (KetaVet, 0.10 mL of 100 mg/mL solution, intramuscularly, Henry Schein) and euthanized with pentobarbital sodium (Dolethal, 1 mL of 200 mg/mL solution, intravenously, Merial Animal Health). A transcardial perfusion was performed (500 mL of 0.1 M phosphate-buffered saline, followed by ~500 mL of 10% formalin fixative). The brain was carefully removed and kept in 10% formalin for a further 24 h and later transferred to phosphate-buffered saline azide solution (0.01 M) for storage. When ready for sectioning, the brain was transferred to 30% w/v sucrose solution for at least 48 h (until the brain sank within the solution, confirming cryoprotection). The tissue was sectioned into 40–60 μm coronal slices on a freezing microtome. The tissues were mounted onto gelatin-coated glass slides and stained with cresyl violet. The placement of cannulae was determined on the basis of tissue damage by examining the mounted brain sections. The placements were schematized by drawing them on coronal sections of the marmoset brain based on the (Paxinos et al. 2012) marmoset brain atlas (Fig. 1*A*). All implants into the aOFC (Fig. 1*B*) and pOFC (Fig. 1*C*) were confirmed to target the intended OFC subregion.

### Statistical Analysis

#### For All Statistical Analyses

All data were subject to tests of sphericity (Mauchly’s test in SPSS) and normality (Shapiro–Wilk test in SPSS). In the event that the condition of sphericity was not met, the Greenhouse-Geisser correction was applied. In the event that the condition of normality was not met, the following steps were taken: All pairwise comparisons were conducted using the Wilcoxon test for nonparametric data; all 1-way analyses involving more than 2 samples were conducted using the Friedman test for nonparametric data; all 2- or 3-way analyses were conducted using the aligned rank transform with the ARTool package in R (Wobbrock et al. 2011). Wherever post hoc pairwise comparisons were conducted, the Sidak correction was applied in SPSS.

#### For the Human Intruder Test

The analyses were separately conducted for the EFA score and all of the component behaviors. The behavioral measures were analyzed using a 2-way linear mixed-effects model analysis (area × treatment; subject as random factor) to account for partially overlapping subjects within the aOFC and pOFC samples.

#### For Basal Cardiovascular Activity in an Affectively Neutral Setting

All measures of cardiovascular activity were analyzed separately. The data were subject to a 2-way linear mixed-effects model analysis (area × treatment; subject as random factor) to account for partially overlapping subjects within the aOFC and pOFC samples.

#### For Discriminative Conditioning to a Proximal Threat

The analyses for CS-directed HR and vigilant scanning behavior were conducted separately. Data for each measure were subjected to a 3-way repeated measures ANOVA (area × treatment × CS trial). Additionally, US^+^-directed cardiovascular responses were analyzed using a 2-way repeated measures ANOVA (area × treatment). The US^−^ was only 0.5 s in duration and therefore did not provide sufficient cardiovascular data for comparative analysis.

#### For Discriminative Conditioning for Reward

The analyses for CS-directed sysBP and head jerks were conducted separately. For the main analysis, CS^−^ responses were subtracted from CS^+^ responses, as previously (Stawicka et al. 2020). The data were analyzed using a 2-way linear mixed-effects model analysis to account for partially overlapping aOFC and pOFC subject groups (area × treatment; subject as random factor). Additionally, the data for individual CS trials were also analyzed in a 3-way linear mixed-effects model analysis (area × treatment × CS), included in the Supplementary Material. The US-directed sysBP responses as a difference between the US^+^ and the US^−^, as well as the amount of marshmallow consumed, were also analyzed using a 2-way linear mixed-effects model analysis (area × treatment; subject as random factor).

#### Inter-rater Reliability Analysis for Behavioral Measures Derived from Videos

For all the key behavioral measures from videos used in the experiments, an inter-rater reliability analysis was conducted on a subset of 6 videos, 2 from each of 3 marmosets. The subset of videos was analyzed by a second rater blind to the conditions. The results were compared using an inter-rater reliability analysis using a 2-way mixed effects model in SPSS, with all results summarized in Supplementary Table 1.

## Results

### Inactivation of the aOFC and pOFC Increases Anxiety-Like Responses to a Distal Threat in the Human Intruder Test

**Figure 2:**
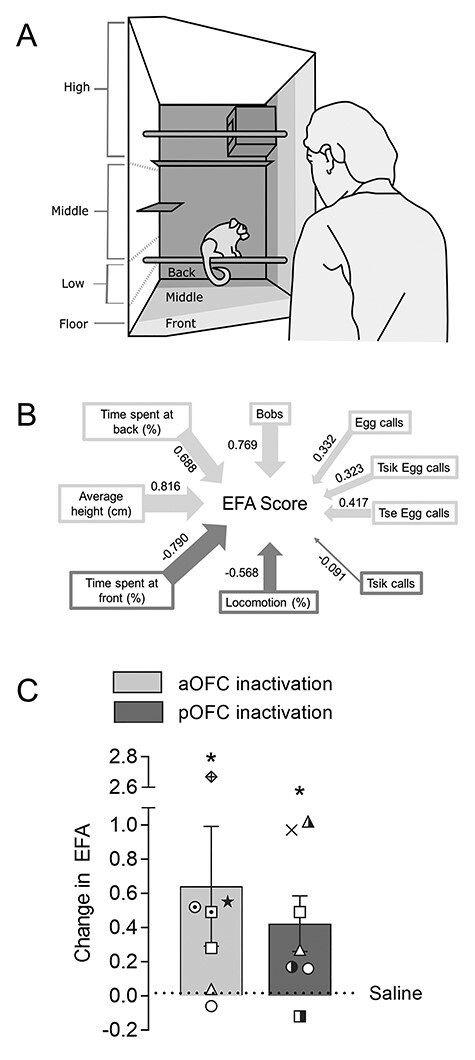
Inactivation of either aOFC or pOFC enhances anxiety-like responses to a distal threat in the human intruder test. (*A*) Schematic showing the confrontation of the subject with an unknown human intruder, along with the subdivisions of the cage used to track the position of the animal. (*B*) Diagram showing the behaviors contributing to the composite EFA score. The thickness of the arrows corresponds to the strength of the contribution, with light gray and dark gray arrows corresponding to positively and negatively contributing behaviors, respectively. (*C*) Graph showing the effects of aOFC (light gray) and pOFC (dark gray) inactivation via Mus-Bac infusion on the EFA score relative to the saline control (line at *y* = 0; data presented as a difference score for clarity). Bars represent the mean, with error bars representing the standard error of the mean. Individual data points for each animal are also presented, with reference to Table 1 (*n* = 7 for both aOFC and pOFC). Significance symbols: ^*^P < 0.05, pairwise comparisons (treatment) for aOFC and pOFC.

Inactivation of the aOFC and pOFC on the human intruder test (Fig. 2*A*) both caused an increase in the EFA score (Fig. 2*C*), representative of a range of different anxiety-like behaviors (Fig. 2*B*), relative to the saline control. A 2-way linear mixed-effects analysis (area × treatment) on the EFA revealed a significant effect of treatment (*F*_(1,14.58)_ = 8.76, *P* = 0.010), with no other effects (area: *F* < 1; area × treatment: *F* < 1). Pairwise comparisons comparing inactivation to control for both aOFC and pOFC confirmed that the effect of inactivation was present for both areas (aOFC saline vs. Mus-Bac: *P* = 0.047; pOFC saline vs. mus-bac: *P* = 0.040). Inactivation of the pOFC was also associated with a consistent increase in average height (Supplementary Table 2). There were no significant effects of aOFC inactivation on any of the individual measures. There was no evidence of significant habituation with repeated tests in animals cannulated in both the aOFC and pOFC. A paired *t*-test on the EFA score for the 2 saline sessions, analyzed according to session order rather than target area, did not reveal a significant difference across repeated sessions (*t* = 1.41, *P* = 0.295).

### Neither Inactivation nor Overactivation of the aOFC or pOFC Alters Basal Cardiovascular Activity in an Affectively Neutral Setting

**Figure 3:**
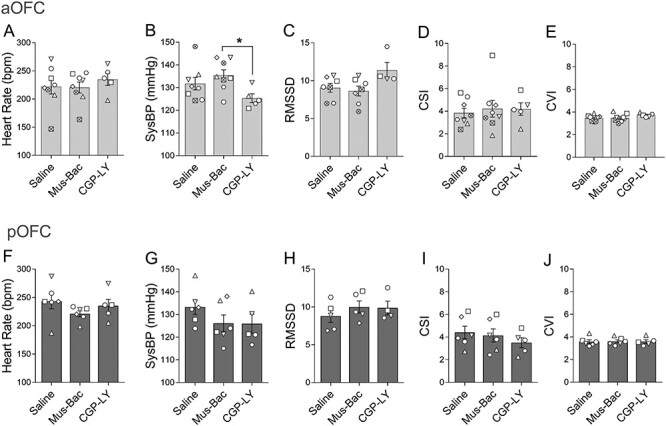
Inactivation or overactivation of aOFC and pOFC has negligible effects on basal cardiovascular activity in an affectively neutral setting. The data are presented separately for the aOFC (top, *A*–*E*) and the pOFC (bottom, *F*–*J*). Effects of inactivation (Mus-Bac) and overactivation (CGP-LY) are compared with the control (Saline) for a number of measures of cardiovascular activity, including HR (*A*, *F*), sysBP (*B*, *G*), HRV (RMSSD [*C*, *H*], CSI [*D*, *I*], and CVI [*E*, *J*]). The bars represent the mean, with error bars denoting the standard error of the mean. Individual data points for each animal are also presented (in reference to Table 1; aOFC saline and Mus-Bac: *n* = 8; aOFC CGP-LY: *n* = 5; pOFC saline and Mus-Bac: *n* = 5; pOFC CGP-LY: *n* = 4). Significance symbols: ^*^  *P* < 0.05, pairwise comparisons (treatment) with Sidak correction, post hoc for linear mixed-effects model analysis (area × treatment).

Separate inactivation or overactivation of aOFC and pOFC did not significantly affect cardiovascular activity in an affectively neutral setting when compared with saline, as measured by HR and sysBP, as well as measures of HR variability including RMSSD, CSI, and CVI (Fig. 3). The 2-way linear mixed-effects analysis revealed a trend toward an effect of inactivation on HR (treatment: *F*_(2,24.83)_ = 3.17, *P* = 0.059; area: *F* < 1; area × treatment: *F*_(2,24.78)_ = 2.33, *P* = 0.118). There were no significant effects for RMSSD (treatment: *F*_(2,20.35)_ = 2.10, *P* = 0.148; area: *F* < 1; area × treatment: *F*_(2,19.42)_ = 1.76, *P* = 0.198), CSI (treatment: *F* < 1; area: *F* < 1; area × treatment: *F*_(2,25.29)_ = 1.23, *P* = 0.309), or CVI (treatment: *F* < 1; area: *F* < 1; area × treatment: *F*_(2,25.02)_ = 1.26, *P* = 0.301). A ratio of CSI to CVI was also analyzed but did not reveal significant effects (treatment: *F* < 1, area: *F* < 1; treatment × area: *F*_(2,20.13)_ = 1.70, *P* = 0.207). For sysBP, there was a significant area by treatment interaction (area × treatment: *F*_(1,24.95)_ = 4.23, *P* = 0.026; area: *F*_(1,25.71)_ = 1.16, *P* = 0.291; treatment: *F*_(2,25.17)_ = 3.17, *P* = 0.055). However, post hoc pairwise comparisons did not reveal any significant effects of either treatment when compared with saline (aOFC: saline vs. Mus-Bac: *P* = 0.419; saline vs. CGP-LY: *P* = 0.145; pOFC: saline vs. Mus-Bac: *P* = 0.072; saline vs. CGP-LY: *P* = 0.145), only between CGP-LY and Mus-Bac for aOFC (CGP-LY vs. Mus-Bac aOFC: *P* = 0.030; pOFC: *P* = 0.995). All of the above data were collected after marmosets showed evidence of successful habituation and stable cardiovascular activity (Supplementary Fig. 1; mean number of sessions of habituation was 13.00, σ = 4.97).

### In contrast to the heightened responsivity to distal threat induced by inactivation of either aOFC or pOFC, aOFC inactivation significantly reduced anticipatory behavioral arousal to proximal threat while pOFC inactivation had no effect

**Figure 4:**
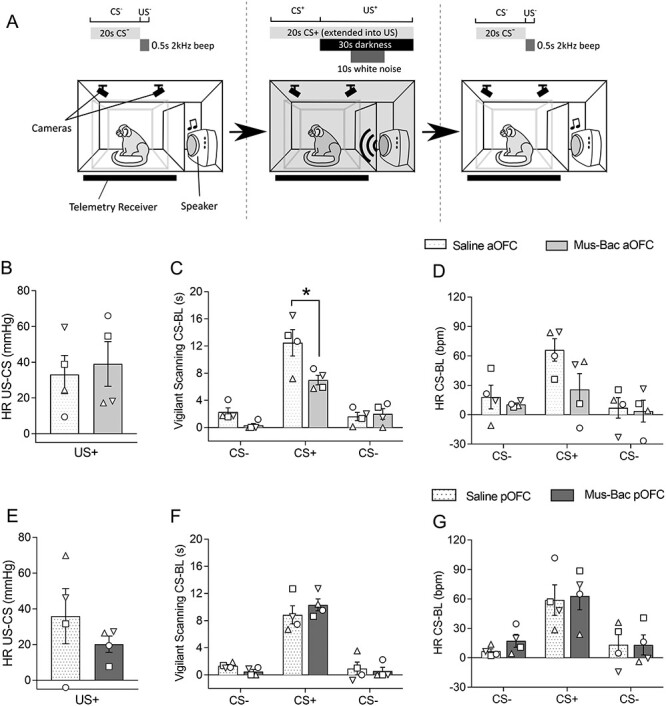
aOFC inactivation reduces conditioned behavioral responses to threat, while pOFC inactivation leaves the responses unchanged. (*A*) Schematic illustrating the apparatus and the 3 CS trials in an infusion session. Effects of inactivation (Mus-Bac) are compared with the control (Saline) for both aOFC (top, light gray) and pOFC (bottom, dark gray). The results are shown for US-directed HR responses (*B*, *E*) where there were no effects. CS-directed behavioral arousal (vigilant scanning; *C*, *F*), as well as HR (*D*, *G*), is also presented. CS-directed effects are shown for each CS, in the order of presentation from left to right across the *x*-axis. The bars represent the mean, with error bars representing the standard error of the mean. Individual data points are presented for each subject, in reference to Table 1 (*n* = 4 for all). Significance symbols: ^*^P < 0.05, pairwise comparisons (treatment) with Sidak correction, post hoc for 3-way repeated measures ANOVA (area × treatment × CS type).

In a discriminative threat conditioning paradigm where a CS^+^ was associated with an imminent aversive event (Fig. 4*A*), all subjects showed successful conditioning, demonstrating increases in HR and vigilant scanning to the CS^+^ but not to the CS^−^ (Supplementary Fig. 2*A*,*B*). They also showed increases in HR, over and above that seen during the CS^+^, in the subsequent US phase, in which the house light was turned off and white noise was presented unpredictably at the start, middle, or end of darkness (Supplementary Fig. 2*C*). The mean number of sessions to acquire discriminative conditioning was 17.00 (σ = 4.97).

Neither inactivation of the aOFC or pOFC affected responsivity to the US^+^ per se, as measured by HR (Fig. 4*B*,*E*). In contrast, while conditioned anticipatory arousal was still present following inactivation of either the aOFC or pOFC, inactivation of aOFC reduced this arousal, although only the reduction in behavior was significant (compare Fig. 4*C* and Fig. 4*D*). Meanwhile, pOFC inactivation did not produce any consistent effects on CS^+^-directed behavioral or cardiovascular arousal (Fig. 4*F*,*G*).

A 2-way ANOVA (area × treatment) on the cardiovascular responses to the US^+^ relative to the preceding CS^+^ (HR US^+^-CS^+^) revealed no significant effects of treatment (*F* < 1) or treatment by area interaction (*F*_(1,3)_ = 2.15, *P* = 0.238).

A 3-way ANOVA (area × treatment × CS) on CS-directed behavioral responses revealed a significant 3-way interaction (area × treatment × CS: *F*_(2,6)_ = 24.27, *P* = 0.001; area × treatment: *F*_(1,3)_ = 8.19, *P* = 0.064; treatment × CS: *F*_(2,6)_ = 1.69, *P* = 0.262; treatment: *F*_(1,3)_ = 7.02, *P* = 0.077; CS: *F*_(2,6)_ = 47.61, *P* < 0.001). Post hoc pairwise comparisons revealed a significant reduction of CS^+^-directed responses following aOFC inactivation compared with saline (*P* = 0.033), with no effect on the CS^−^ (first: *P* = 0.125; second: *P* = 0.479). Despite this, CS^+^-directed responses were still significantly higher than those to the CS^−^ (first CS^−^: *P* = 0.012; second CS^−^: *P* = 0.053), although blunted compared with saline (first CS^−^: *P* = 0.001; second CS^−^: *P* < 0.001). They were also significantly above their preceding baseline (aOFC saline: *P* = 0.007; aOFC Mus-Bac: *P* = 0.004). In contrast, there were no significant effects of inactivation of pOFC (first CS^−^: *P* = 0.159; CS^+^: *P* = 0.495; second CS^−^: *P* = 0.779).

For HR, there was no significant 3-way interaction on the ANOVA (area × treatment × CS; *F*_(2,6)_ = 3.02, *P* = 0.124), with only a significant effect of CS (CS: *F*_(2,6)_ = 21.16, *P* = 0.002; treatment × CS:*F*_(2,6)_ = 1.01, *P* = 0.418; area × treatment: *F*_(1,3)_ = 2.75, *P* = 0.196; treatment: *F* < 1). However, it can be seen that following aOFC inactivation there was a consistent reduction in HR that mirrored that seen in behavior. When the effects on the CS^+^-directed HR were considered separately using a 2-way repeated measures ANOVA (area × treatment), there was a significant interaction (area × treatment: *F*_(1,3)_ = 29.06, *P* = 0.013; area: *F* < 1; treatment: *F*_(1,3)_ = 1.94, *P* = 0.258), with pairwise comparisons confirming a significant effect of aOFC inactivation compared with the control (*P* = 0.037), but no effect with pOFC inactivation (*P* = 0.814).

There was no evidence of significant habituation in performance, based on the US^+^-directed and CS^+^-directed HR responses across the course of the Pavlovian threat discrimination study. The analysis of the 2 saline sessions (aOFC and pOFC), based on the order of occurrence rather than target area, did not reveal a significant effect of order (2-way repeated measures ANOVA on CS-directed responses [session order × CS type]—session order: *F* < 1, CS type: *F*_(2,6)_ = 11.26, *P* = 0.009, session order by CS type: *F* < 1; US^+^-directed HR responses, saline 1 vs. saline 2 paired *t*-test—*t* = 1.50, *P* = 0.231).

### Inactivation of the pOFC Has No Effects on the Recovery of Arousal Following US ^+^ Omission

**Figure 5:**
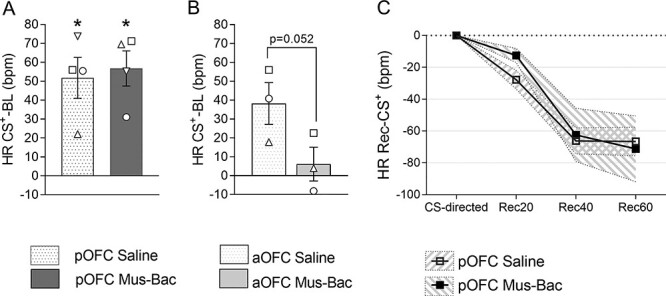
pOFC inactivation does not affect recovery of conditioned cardiovascular responses to threat with US^+^ omission. (*A*) A comparison of CS^+^-directed HR responses (CS-BL) during pOFC control and inactivation sessions, showing comparable levels of arousal prior to recovery. (*B*) A comparison of CS^+^-directed HR responses during aOFC control and inactivation sessions, showing a reduction in CS^+^-directed responses with inactivation. The bars represent the mean, with error bars representing the standard error of the mean. Individual data points are presented for each subject, in reference to Table 1. (*C*) Recovery of CS^+^-directed HR arousal across a 60-s recovery period split into 3 bins (Rec20, Rec40, Rec60). The CS-directed response is presented for reference and was not included in the analysis of recovery. Data are compared for the pOFC control (saline, empty squares) and inactivation (Mus-Bac, filled squares). Each point represents the mean HR relative to the CS^+^-directed response, with the shaded region representing the standard error of the mean (*n* = 4 for *A* and *C*, *n* = 3 for *B*). Significance symbols: ^*^P < 0.05, one-sample *t*-tests comparing CS^+^-directed HR to 0, with Sidak correction for multiple tests (Saline and Mus-Bac).

In the absence of effects of pOFC inactivation on the expression of threat-elicited conditioned responses, possible regulation of these responses was examined by testing the rate of recovery of cardiovascular arousal back to baseline following the presentation of the CS^+^ in the absence of the US^+^ (as investigated following excitotoxic lesions in Reekie et al. 2008). As expected, there was no effect of pOFC inactivation on the CS-directed responses per se (Fig. 5*A*), but there was also no effect on the subsequent recovery of the HR response, which declined rapidly following the termination of the CS^+^ at a rate comparable to the control (Fig. 5*C*). The effects of aOFC inactivation were not tested due to the blunting of CS^+^-directed arousal associated with the treatment, replicated in this experiment (Fig. 5*B*).

Preliminary analyses on 2 control sessions (saline) revealed that HR responses showed a consistent decline following CS^+^ termination across marmosets and that this decline was stable across repeated control sessions (Supplementary  Fig. 3).

Effects of pOFC inactivation on HR recovery were analyzed using a 2-way repeated measures ANOVA (treatment × phase), where the 60-s recovery period was split into three 20-s recovery bins (phase: Rec20, Rec40, Rec60) and normalized to the CS-directed response. The analysis revealed a trend toward an effect of phase (*F*_(2,6)_ = 8.65, *P* = 0.060) and no treatment by phase interaction (*F*_(2,6)_ = 3.80, *P* = 0.145) and no treatment effect (*F* < 1).

CS^+^-directed HR responses were initially analyzed for both aOFC and pOFC with a 2-way linear mixed-effects model analysis (area × treatment), to identify any effects on the response which could affect subsequent recovery. The analysis revealed a significant effect of area (*F*_(1,8.15)_ = 10.93, *P* = 0.010), a trend toward an area by treatment interaction (*F*_(1,7.27)_ = 4.11, *P* = 0.081) and no main effect of treatment (*F*_(1,7.27)_ = 2.22, *P* = 0.179). Post hoc pairwise comparisons revealed no effect of pOFC inactivation on CS-directed responses (pOFC saline vs. Mus-Bac: *P* = 0.692), but a clear trend toward a reduction of responses with aOFC inactivation, similar to that seen on the standard task (aOFC saline vs. Mus-Bac: *P* = 0.052). A single-sample *t*-test against 0 revealed that the HR increase above baseline was significant for both the pOFC control (*P* = 0.018) and inactivation (*P* = 0.018).

### Inactivation of the aOFC Reduced Anticipatory Cardiovascular Responses to Conditioned Reward, with no Effects of pOFC Inactivation

**Figure 6:**
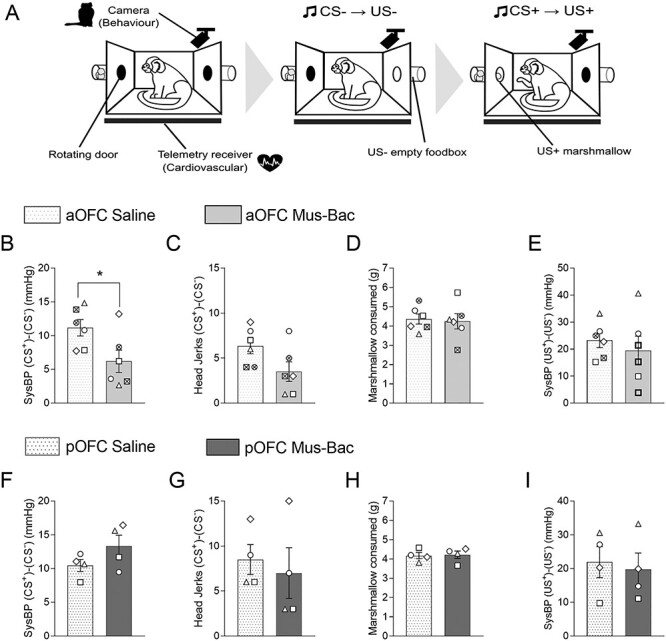
aOFC inactivation reduces appetitive CS^+^-directed appetitive cardiovascular arousal relative to the CS^−^, while pOFC inactivation leaves the responses unchanged. (*A*) Schematic illustrating the apparatus and the CS^−^ and CS+ trials in an infusion session. Effects of inactivation (Mus-Bac) are compared with the control (Saline) for both aOFC (top, *B*–*E*) and pOFC (bottom *F*–*I*). The effects are compared on CS-directed sysBP changes (*B*, *F*), as well as behavior (appetitive head jerks: *C*, *G*). CS-directed effects are presented as the difference between CS^+^ and CS^−^-directed responses. The results are also shown for US-directed responses for marshmallow consumption (*D*, *H*) and sysBP (*E*, *I*). The bars represent the mean, with error bars representing the standard error of the mean. Individual data points are presented for each subject, in reference to Table 1 (*n* = 6 for aOFC, *n* = 4 for pOFC). Significance symbols: ^*^P < 0.05, pairwise comparisons (treatment) with Sidak correction, post hoc for linear mixed effects model analysis (area × treatment).

Marmosets were conditioned to discriminate between 2 neutral CS, where the CS^−^ predicted 2 min of a visible empty food box and the CS^+^ predicted 2 min of access to a food box filled with marshmallow (Fig. 6*A*). All subjects showed evidence of successful cardiovascular and behavioral conditioning, showing heightened responses to the CS^+^ relative to the CS^−^ following training and immediately prior to the start of drug treatment (Supplementary Fig. 4). They also showed increases in blood pressure during the consumption of reward, indicative of the arousing nature of the stimulus. The mean number of sessions to acquire appetitive discriminative conditioning was 29.17 (σ = 5.12).

On the test day, marmosets were presented with a CS^−^ trial followed by a CS^+^ trial. Inactivation of the aOFC resulted in a significant reduction in CS^+^-directed blood pressure responses. This was evident by looking both at the CS^+^-directed response relative to the CS^−^ (Fig. 6*B*), as well as the CS-directed responses analyzed independently (Supplementary Fig. 5A). Inactivation of the aOFC was also associated with a reduced behavioral response to the CS^+^ in most marmosets, although this did not reach statistical significance (Fig. 6*C*). Despite the effects of aOFC inactivation on conditioned responses, there were no significant effects on unconditioned responses as measured by consumption of marshmallow (Fig. 6*D*) and the associated cardiovascular arousal (Fig. 6*E*). Inactivation of the pOFC produced no effects on conditioned or unconditioned measures (Fig. 6*F*–*I*).

A 2-way linear mixed-effects model analysis (area × treatment; subject as random factor) of the CS^+^-CS^−^ blood pressure responses revealed a significant area by treatment interaction (treatment × area: *F*_(1,12.31)_ = 6.75, *P* = 0.023). Post hoc pairwise comparisons revealed a significant reduction following aOFC inactivation relative to its control (aOFC saline vs. Mus-Bac: *P* = 0.022), with no effect of pOFC inactivation (pOFC saline vs. Mus-Bac: *P* = 0.244). The same analysis on head jerk behavior revealed only a trend toward an effect of treatment (treatment × area: *F* < 1; treatment: *F*_(1,11.64)_ = 0.093; area: *F*_(1,12.99)_ = 4.24, *P* = 0.060). In addition, there were no significant effects with respect to the US for either blood pressure (treatment: *F*_(1,11.04)_ = 2.19, *P* = 0.167; treatment × area: *F* < 1) or marshmallow consumption (treatment: *F* < 1; treatment × area: *F* < 1). The responses to the CS^−^ and CS^+^ individually were also analyzed, revealing equivalent effects (Supplementary Fig. 5).

There was no evidence of significant habituation in performance across the course of the Pavlovian appetitive discrimination study, based on the US^+^-directed and CS^+^-directed sysBP responses across time. The analysis of the 2 saline sessions (aOFC and pOFC, where available), based on the order of occurrence rather than target area, did not reveal a significant effect of order (2-way repeated measures ANOVA on CS-directed responses across saline sessions [session order × CS type]—session order: *F* < 1; CS type: *F*_(1,3)_ = 93.27, *P* = 0.002; session order × CS type: *F* < 1; 2-way repeated measures ANOVA on US-directed responses across saline sessions [order × US type]—order *F* < 1; US type *F*_(1,3)_ = 31.25, *P* = 0.011; order × US type *F*(1,3) = 7.35, *P* = 0.073).

### The Blunting of CS ^+^-Directed Blood Pressure Responses with aOFC Inactivation Is Limited to Sessions with a Preceding CS ^−^

**Figure 7:**
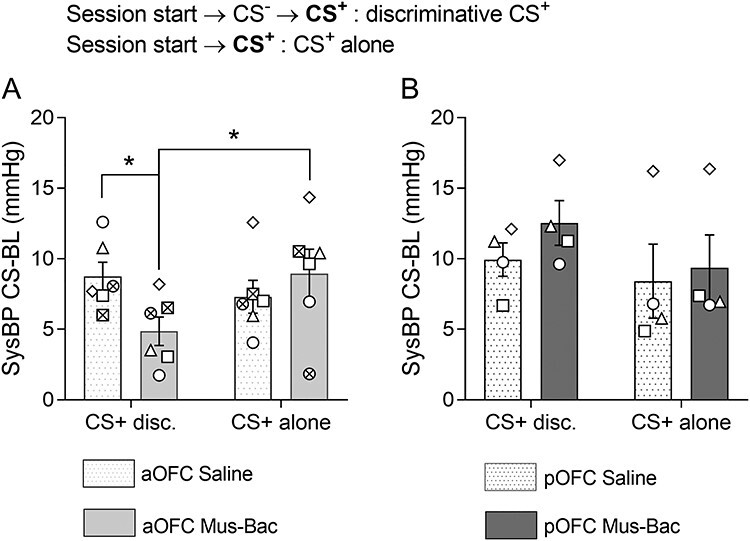
Inactivation of the aOFC reduces sysBP appetitive CS^+^-directed responses on trials preceded by a CS^−^, but not single CS^+^ trials. The graph presents the CS^+^-BL sysBP responses after Mus-Bac and saline infusions, comparing them between sessions with a CS^−^ trial preceding the CS^+^ trial (i.e., discriminative, CS^+^ disc.) and sessions with a single CS^+^ trial (CS^+^ alone). The data are presented for both aOFC inactivation (*A*) and pOFC inactivation (*B*). The bars represent the mean, with error bars representing the standard error of the mean. Individual data points are presented for each subject, in reference to Table 1 (*n* = 6 for aOFC, *n* = 4 for pOFC). Significance symbols: ^*^ is *P* < 0.05, pairwise comparisons (treatment and session type) with Sidak correction, post hoc for linear mixed-effects model analysis (area × treatment × session type).

Marmosets were finally tested on a session designed to determine the effects of inactivation on the recovery of appetitive arousal in the event of US^+^ omission (CS^+^ presented without the expected outcome). This involved presentation of a single CS^+^ trial. Unexpectedly, inactivation of the aOFC did not produce the expected blunting of CS^+^-directed responses previously observed in sessions with both trial types (Fig. 7*A*). Seeing as marmosets were familiar with single CS^+^ sessions, and at the point when the CS^+^ was presented the marmosets could not have anticipated the absence of the US^+^, the only possible explanation for this distinction is the absence of a preceding CS^−^. Inactivation of the pOFC did not produce any notable effects (Fig. 7*B*).

The comparison of CS^+^-directed responses in the 2 different session types (with or without a CS^−^) was conducted using a 3-way linear mixed-effects model analysis (area × treatment × session type; subject as random factor). The analysis revealed a significant 3-way interaction between area, treatment, and session type (area × treatment × session type: *F*_(1,27.36)_ = 4.58, *P* = 0.041; area × treatment: *F*_(1,27.36)_ = 2.97, *P* = 0.096; treatment × session type: *F*_(1,27.36)_ = 1.33, *P* = 0.260; treatment: *F* < 1; session type: *F* < 1). Importantly, the analysis revealed that there was a significant difference in the effects of inactivation when the CS^+^ was presented following a CS^−^ trial and when it was presented alone (Mus-Bac discriminative vs. Mus-Bac alone: *P* = 0.011). Additional post hoc pairwise comparisons confirmed the significant difference between CS^+^-directed responses following aOFC inactivation compared with the aOFC control in discriminative test sessions (*P* = 0.016), but showed no effect of inactivation in sessions with the CS^+^ alone (*P* = 0.284). In contrast, consistent with no effects of pOFC inactivation on the standard task, there were no effects of pOFC inactivation on the CS^+^-directed responses compared with the pOFC control in either session type (discriminative: *P* = 0.169, alone: *P* = 0.613).

### Inactivation of the aOFC, but Not pOFC, Accelerated the Recovery of Appetitive Conditioned sysBP Responses in the Absence of a US ^+^

**Figure 8:**
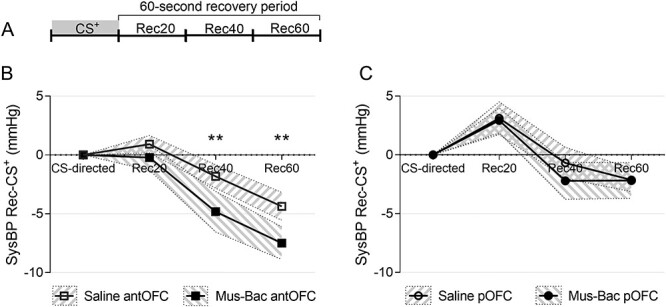
Effects of aOFC and pOFC inactivation of recovery of appetitive conditioned arousal in the absence of the US^+^. (*A*) A schematic showing the structure of the probe CS^+^ trial and the subdivisions of the recovery period used for analysis. The recovery of CS^+^-directed sysBP arousal is presented separately for aOFC inactivation (*B*, square points) and pOFC inactivation (*C*, circular points). The CS-directed response is presented for reference and was not included in the analysis of recovery. Each point represents the mean. The shaded area surrounding the points represents the standard error. *n* = 6 for *B* and *n* = 4 for *C*. Significance symbols: ^*^^*^P < 0.01, pairwise comparisons (treatment) with Sidak correction, post hoc for linear mixed-effects model analysis (area × treatment × recovery phase).

The discovery that CS^+^-directed responses were not affected in the single trial sessions and were equivalent following saline and inactivation infusions allowed for the comparison of both aOFC and pOFC inactivation effects on the recovery of appetitive conditioned responses with US^+^ omission. Inactivation of the aOFC appeared to accelerate the recovery of CS^+^-directed sysBP responses (Fig. 8*B*), while inactivation of the pOFC did not (Fig. 8*C*). Any observed effects were not due to the order in which treatment took place (Supplementary Fig. 6).

A 3-way linear mixed-effects model analysis (area × treatment × recovery phase) on sysBP recovery revealed significant effects of treatment (*F*_(1,43.12)_ = 9.16, *P* = 0.004), area (*F*_(1,44.68)_ = 28.93, *P* < 0.001), recovery phase (*F*_(1,43.12_ = 46.94, *P* < 0.001), and no other effects (area × treatment: *F*_(1,43.12)_ = 3.40, *P* = 0.072; area × phase: *F*_(2,43.12)_ = 1.22, *P* = 0.305; treatment × phase: *F* < 1; area × treatment × phase: *F* < 1). Post hoc pairwise comparisons revealed that the effect of treatment was primarily attributable to the aOFC, with no effects in the pOFC. There were significant effects of aOFC inactivation on recovery in the later phases (saline vs. Mus-Bac, Rec40: *P* = 0.009, Rec60: *P* = 0.006) but not the pOFC (saline vs. Mus-Bac, Rec20: *P* = 0.887, Rec40: *P* = 0.258, Rec60: *P* = 0.975).

## Discussion

The present study shows that temporary, pharmacological inactivation of either the aOFC or pOFC heightens responses to a more distal, uncertain threat but not to a proximal, certain threat. Distal threat reactivity was measured in response to an unknown human that tends to elicit conflicting motivations to either approach or withdraw (described in Agustín-Pavón et al. 2012). Both aOFC and pOFC inactivation produced an anxiety-like effect. However, neither manipulation heightened conditioned responses to an imminent aversive stimulus (darkness and aversive white noise) known to elicit cardiovascular and behavioral arousal in marmosets (Alexander et al. 2020). Inactivation of the pOFC had no effects on the expression of such Pavlovian threat responses, while perhaps surprisingly, aOFC inactivation blunted behavioral conditioned responses to threat. A similar blunting of the conditioned response was also seen in the appetitive version of Pavlovian conditioning following aOFC inactivation, suggesting a common underlying mechanism by which aOFC inactivation disrupted the expression of discriminative conditioning, independent of valence. Moreover, the finding that this blunting was only present when the CS^+^ was preceded by a CS^−^, but not when the CS^+^ was presented alone in the appetitive context, highlights further the likely cognitive, rather than affective, nature of the deficit. Due to a link between cardiovascular health and affective disorders, effects of aOFC and pOFC inactivation and overactivation were also tested on basal cardiovascular activity, with no effect.

Experiments manipulating threat imminence in humans have suggested that the presence of more distal, uncertain threat engages the PFC, in a way which is not observed in response to a proximal and more imminent threat (Mobbs et al. 2020). It has been argued that the time available when encountering distal threat allows for the engagement of prefrontal-dependent processes, including attentional, appraisal, and strategizing, that are less appropriate when the threat is imminent and rapid responses are required. In support of this proposal, we recently showed that temporary manipulations within medial orbital area 14 of the marmoset primarily affected distal threat in the form of the human intruder but not responsivity to proximal threat as measured by the expression of Pavlovian threat conditioning (Stawicka et al. 2020). We have shown a similar dissociation with excitotoxic lesions of the marmoset aOFC (Agustín-Pavón et al. 2012). The present study extends these findings to reveal that heightened reactivity to distal but not proximal threat is not only seen following temporary inactivation of aOFC (as opposed to permanent lesions) but also as a consequence of temporary inactivation of pOFC. It should be noted though that there were differences in the reactivity of marmosets with permanent lesions and temporary inactivation of aOFC. The former resulted not only in increased distance, and hence withdrawal, from the intruder, but also increases in head and body bobs and vocalizations associated with vigilance (Agustín-Pavón et al. 2012). In contrast, the latter while also inducing withdrawal did not increase the species-specific behaviors, which remained unchanged. These differences could reflect the distinction between the effects of acute inactivation producing an anxiety-like state, versus permanent manipulations, possibly associated with a reorganization of circuits, leading to the development of an anxious-like trait with all the associated behaviors that accompany such traits (see Shiba et al. 2015 and Quah et al. 2020 for a discussion of traits).

In contrast to aOFC, pOFC inactivation had not been previously examined on threat reactivity. However, it might have been surmised that given the pOFC’s strong connectivity with the amygdala (Timbie and Barbas 2015) and the finding that lesions of the amygdala have been shown to reduce responsivity to the human intruder in macaques (Kalin et al. 2004), that pOFC inactivation may also reduce threat reactivity. In contrast, however, pOFC inactivation heightened reactivity, similar in extent and patterning of behavior to the aOFC.

These effects of heightened reactivity to threat following inactivation of central regions of OFC in the marmoset stand in contrast to the blunting of threat reactivity to the human intruder following large OFC ablations in macaque monkeys (Kalin et al. 2007; Machado and Bachevalier 2008; see Oikonomidis et al. 2017 for a review of this literature) that variably removed either areas 11, 13, and 14 (Machado and Bachevalier 2008) or the entire orbital surface, which also included orbital 12 *(*Kalin et al. 2007). However, ablations have major effects on regions distal to the ablated region due to the disruption of fibers of passage, and thus, effects seen may or may not be due to loss of the targeted brain region (see Murray and Rudebeck 2018 for a review). In contrast, a more recent study using selective excitotoxins to permanently lesion central OFC in the macaque (areas 11 and 13) revealed a similar heightening of threat reactivity in macaques, as identified in marmosets, when reaching for reward in the presence of a rubber snake (Pujara et al. 2019). Where area 13, within pOFC, has been selectively targeted in the macaque, a reduction in attention toward the eyes of conspecifics displaying aggression (negative social stimulus), has been reported, which the authors interpreted as reflecting reduced social anxiety (Murphy and Bachevalier 2020). However, there are a number of caveats in this study worth considering, including the relatively small and variable extent of the lesion within area 13 across individuals and an alternative explanation for the findings which suggests that less time focusing on the eyes may be a manifestation of increased social anxiety.

Although aOFC and pOFC inactivation both heightened reactivity to distal threat, these effects did not extend to the conditioned responses associated with proximal threat. The use of CS predicting an imminent threat, in this case darkness and white noise, has been proposed as a means for studying proximal threat (Mobbs et al. 2020) and the lack of effect of pOFC inactivation rules out this region in regulating the expression of proximal threat-induced conditioned responses. In contrast, not only did inactivation of the aOFC fail to heighten responses, but perhaps more surprisingly, it significantly blunted behavioral conditioned arousal to the CS^+^. This was also associated with consistent trends in the blunting of cardiovascular activity too and contrasts with the lack of effects observed with permanent excitotoxic lesions of the aOFC in the marmoset (Agustín-Pavón et al. 2012). The effect of temporary aOFC inactivation to blunt the conditioned response was not, however, valence specific, as a similar blunting effect was seen after aOFC inactivation on appetitive conditioned responses. Once again, pOFC inactivation was without effect suggesting a selective role for the aOFC. The nature of this effect requires further, future investigation but clues come from the serendipitous finding that the blunting effect was apparently restricted to sessions in which the CS^+^ trial was preceded by a CS^−^ trial. The conditioned responses were unaffected in sessions where the CS^+^ was presented alone; although this was only shown for Pavlovian appetitive discriminative conditioning. Unfortunately, this was not investigated in the context of aversive discriminative conditioning; although future studies should do so. This finding rules out an explanation in terms of selective disruption of appetitive conditioned arousal per se and instead suggests that aOFC inactivation disrupted the discriminative process whereby the expression of conditioned arousal is dependent upon CS specificity. A similar dissociation has been seen previously following anterior cingulate lesions in rats (Cardinal et al. 2003). With respect to the aOFC, its connectivity with areas including ventrolateral and dorsolateral PFC and frontal pole (Carmichael and Price 1996; Cavada et al. 2000) could point toward disruptions of attentional allocation and strategizing, as a consequence of a disconnect between these regions. While the OFC has been proposed to play an important role in encoding task states and their transitions, particularly when based on partially unobservable information (Wilson et al. 2014), a loss of this function is less likely to underlie the observed effects here, since the CS^−^ and CS^+^ were fully observable stimuli.

An important consideration when comparing the effects of manipulations on the human intruder test and Pavlovian discriminative conditioned threat paradigm is that there are more differences between these 2 tests other than threat imminence. The threats were different, being an unknown human versus white noise and darkness and the environment in which the threats were presented also differed, being the home cage versus a small test apparatus. Thus, to further test the threat imminence hypothesis and the specific contribution of the OFC to distal and not proximal threat, it will be important to employ more comparable tests that differ along one psychological dimension only, that is, time, space or probability. For example, we have recently showed that overactivation of medial OFC, area 14, which heightens responsivity to the human intruder but has no effect on the expression or extinction of Pavlovian conditioned threat responses, does however heighten reactivity to the same Pavlovian stimuli, associated with the same threat, in the same test apparatus, when the threat is uncertain (less probable) rather than certain (Stawicka et al. 2020).

A major component of affective disorders is increased morbidity and mortality resulting from cardiovascular disease (Musselman et al. 1998; Batelaan et al. 2016), and recent evidence has implicated subcallosal cingulate area 25, not only in the regulation of affect-induced changes in cardiovascular activity but also at rest (Wallis et al. 2017; Alexander et al. 2020). In contrast, interventions in vmPFC area 14 only affected positive or negative valence-induced changes in cardiovascular activity but had no effect on resting state (Stawicka et al. 2020). Here, too, no effects of inactivation of either the aOFC or pOFC were observed on basal cardiovascular activity. Since past experiments using electrical stimulation in macaques (Hall et al. 1977) and humans (Chapman et al. 1949) have shown that stimulation within the OFC, and in particular the pOFC, resulted in changes in resting HR and blood pressure, the effects of overactivation were also determined; but no such effects were seen in either the aOFC or pOFC. Thus, the effects from these early experiments may have been the result of activation of fibers of passage or were indirect effects due to intense stimulation at levels outside of the physiological range.

Together, these experiments into OFC function have presented a unique profile of effects on threat- and reward-elicited behaviors. The effects of aOFC and pOFC inactivation, primarily encompassing areas 11 and 13, respectively, set them apart from the neighboring medial OFC area 14. While aOFC and pOFC inactivation heighten responses to a distal threat, the same effect is observed following overactivation of area 14 (Stawicka et al. 2020). In all cases, however, the heightening of reactivity to negative stimuli appears restricted to distal but not proximal threat responses, consistent with the proposal that PFC mechanisms are primarily engaged when there is time to attend, appraise, and strategize (Mobbs et al. 2020). This contrasts with neighboring subcallosal area 25, overactivation of which heightens reactivity to negative stimuli in both distal and proximal threat domains (Alexander et al. 2020). The latter is also unique among these regions in that it also regulates basal levels of cardiovascular activity, with overactivation tipping the balance toward sympathetic control. Here, despite pOFC having connections with hypothalamic nuclei (Öngür et al. 1998) implicated in cardiovascular control (Smith et al. 1990), it did not contribute to the regulation of basal cardiovascular activity.

In summary, this study has begun to separate out the contribution of the aOFC and pOFC from neighboring regions in the regulation of distal and proximal threat responses. In particular, the finding that inactivation of aOFC and pOFC, but overactivation of areas 14 and 25, induce an apparently similar high anxiety-like phenotype in response to distal threat highlights the varied neurobiological mechanisms that can underlie anxiety symptoms in humans and which may contribute to the individual variation in responsivity to treatments. We propose that a comparison of distinct classes of anxiolytics on heightened threat reactivity induced by opposing interventions in these different prefrontal and cingulate brain regions will provide important new insights into individualized treatment strategies.

## Supplementary Material

ZStawicka_Supplementary_Final_bhab240
