# Supplementary material for: Differential Effects of the Inactivation of Anterior and Posterior Orbitofrontal Cortex on Affective Responses to Proximal and Distal Threat, and Reward Anticipation in the Common Marmoset
Source: Cereb Cortex. 2021 Sep 8;32(7):1319–36. doi: 10.1093/cercor/bhab240 (PMC8971078; doi:10.1093/cercor/bhab240)
Supplement: ZStawicka_Supplementary_Final_bhab240 [file zstawicka_supplementary_final_bhab240.pdf]

Experiment	Measure	Rater 1	Rater 2	Intraclass Correlation Coefficient	Significance
**Human Intruder Test**	EFA	-0.65 ± 0.27	-0.74 ± 0.22	0.944	**p=0.002****
**Conditioned Responses to Threat**	CS^{-}_{1} -BL	0.85 ± 0.39	0.84 ± 0.37	0.946	**p<0.001*****
	CS^{+} - BL	9.10 ± 2.08	9.42 ± 1.84	0.980	**p<0.001*****
	CS^{-}_{2} - BL	1.35 ± 0.53	1.33 ± 0.47	0.984	**p<0.001*****
**Conditioned Responses to Reward (difference score)**	(CS^{+}-BL)-(CS^{-}-BL)	6.67 ± 1.17	4.5 ± 0.81	0.861	**p=0.025***

Supplementary Table 1 – Inter-rater reliability analysis on key behavioural measures derived from video scores. The key measures include the exploratory factor analysis (EFA) for the human intruder test, the CS-directed vigilant scanning as a measure of conditioned responses to threat, and the difference score of CS-directed appetitive head jerks as a measure of conditioned responses to reward. A subset of six videos from each experiment were scored by a second rater blind to infusion conditions and submitted to an inter-rater reliability analysis. The table displays the results on the intraclass correlation analysis using a two-way mixed effects model in SPSS, including mean results ± the standard error of the mean, the Intraclass Correlation Coefficient and the resulting statistical significance. Significant results indicate agreement between the two raters. Significance symbols: ** p<0.01, *** p<0.001.

Measure	EFA contribution	aOFC Mus-Bac – Saline (Mean ± SEM)	pOFC Mus-Bac – Saline (Mean ± SEM)	aOFC Mus-Bac vs Saline	pOFC Mus-Bac vs Saline
Time spent at the front (%)	-0.790	-17.85 ± 12.24	-2.71 ± 5.50	p=0.195	p=0.640
Time spent at the back (%)	+0.688	26.57 ± 12.96	17.27 ± 8.77	p=0.086	p=0.096
Height (cm)	+0.816	15.52 ± 8.03	10.76 ± 4.23	p=0.102	p=0.044*
Head and body bob	+0.769	0.00 ± 1.81	6.29 ± 6.26	p=0.869	p=0.703
Locomotion (%)	-0.568	2.93 ± 1.94	-1.72 ± 2.62	p=0.182	p=0.535
Tsik calls	-0.091	-2.43 ± 2.32	0.43 ± 0.81	p=0.170	p=0.803
Tsik-egg calls	+0.323	-4.29 ± 3.31	5.14 ± 6.34	p=0.390	p=0.305
Tse-egg calls	+0.417	-4.29 ± 2.23	-3.42 ± 1.73	p=0.058	p=0.121
Egg calls	+0.332	5.14 ± 5.75	-1.00 ± 2.37	p=0.217	p=0.806
Total vigilance calls	-	-3.43 ± 7.12	0.71 ± 7.50	p=0.631	p=0.920

Supplementary Table 2 – Analysis of behaviours contributing to the EFA on the human intruder test. Data is presented for all the contributing behaviours (“Measure”), along with the mean effect of inactivation (presented as the differences score: Mus-Bac – Saline) for aOFC and pOFC. Component behaviours were analysed using two-tailed paired t-tests (or the Wilcoxon test for non-parametric data, verified using the Shapiro-Wilk test) for the aOFC and pOFC separately. Significance symbols: * $p<0.05$, paired two-tailed t-test comparing Saline versus Mus-Bac.

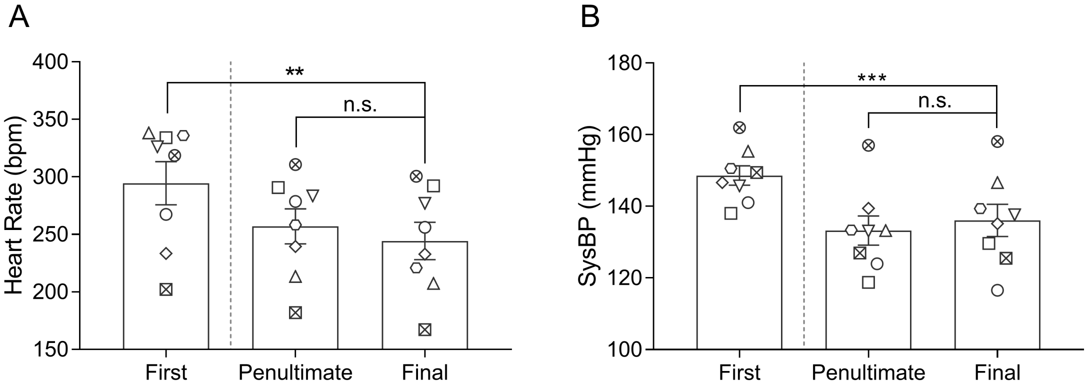

Supplementary Figure 1 – Marmosets successfully habituated to the testing apparatus and showed evidence of stable cardiovascular activity between sessions. The mean HR (A) and sysBP (B) over the entire 20-minute session is shown for the first, penultimate and final session before drug treatment began. There was a statistically significant difference in HR between the 3 sessions (n=8, one-way repeated measures ANOVA, $F_{(2,14)}$=7.23, p=0.007). The post-hoc analysis revealed a significant difference between the first and final session (p=0.007) but not the final and penultimate session (p=0.631). The same pattern was also observed for sysBP with a significant effect of session (n=8, one-way repeated measures ANOVA, $F_{(2,14)}$=21.06, p<0.001), with pairwise comparisons revealing a significant difference between the first and last session (p<0.001) and no significant difference between the last and penultimate session (p=0.515). Bars represent the mean, with error bars representing the standard error of the mean. The individual data points for each animal are also presented as designated in Table 1. Significance symbols: n.s., p>0.05; **, p<0.01; ***, p<0.001, pairwise comparisons (session), post hoc for ANOVA (as above).

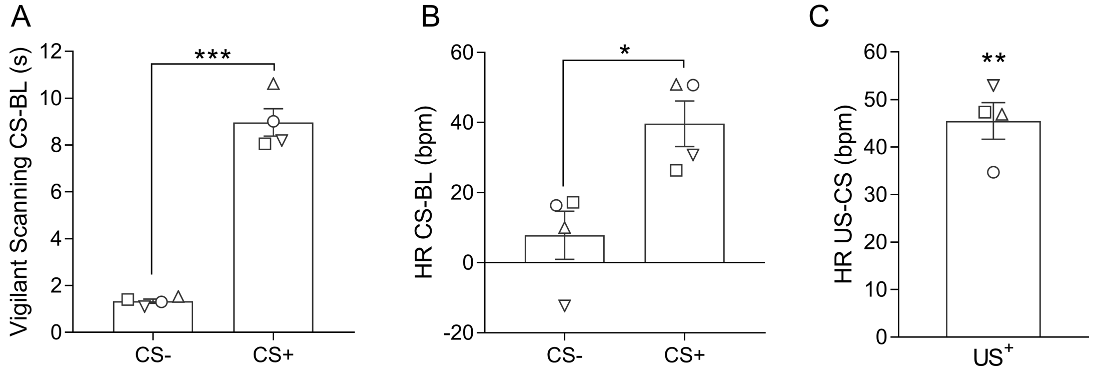

Supplementary Figure 2 – Marmosets show evidence of successful discriminative conditioning to threat. Data presented is the mean of responses of each subject over the 3 discriminative conditioning sessions immediately before drug treatment began. Since the number of CS^- trials varied across session types, the responses to the CS^- for each session were combined as a mean. This is presented for CS-directed behaviour (A) and cardiovascular responses (HR; B) for both CS^- and CS^+. The US^+-directed cardiovascular responses (US-CS HR) are also presented (C), confirming responsiveness to the threat itself. Paired t-tests confirmed a significant difference between the mean CS^+ and CS^--directed responses for both behavioural and cardiovascular measures (CS^+ vs CS^-; behaviour: $t=14.39$, $p<0.001$; HR: $t=4.08$, $p=0.027$). A one-sample two-tailed t-test was also employed to test the mean US^+ response, revealing a significant rise in HR exceeding 0 ($t=11.81$, $p=0.001$). The bars represent the mean, with the error bars representing the standard error of the mean. Individual data points are presented for each animal as designated in Table 1 (n=4 for all). Significance symbols: * is $p<0.05$, ** is $p<0.01$, *** is $p<0.001$, paired t-tests (as above).

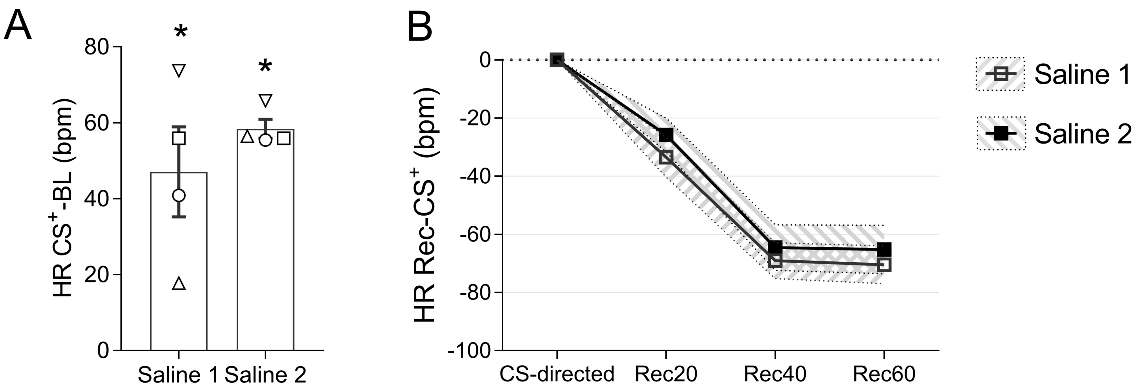

Supplementary Figure 3 – Heart rate showed a consistent and significant recovery following CS$^+$ presentation with US$^+$ omission across repeated control sessions (discriminative conditioning to threat). Graphs present the results from the experiment using a standard CS$^-$/CS$^+$/CS$^-$ session, where the CS$^+$ is presented for 20 seconds without US$^+$ presentation. Data from two saline sessions (aOFC and pOFC) is compared, analysed based on the order in which they took place rather than the target area (there was an inactivation session in-between the two saline sessions). A. presents the CS$^+$-directed HR responses and B demonstrates the recovery of HR responses across the recovery period. The 60-second recovery period is split into 3 bins (Rec20, Rec40, Rec60), with HR presented relative to the CS-directed response. The CS-directed response is presented for reference and was not included in the analysis of recovery. Repeated probe sessions with US$^+$ omission did not significantly affect CS-directed responses: the Wilcoxon test on HR responses showed no significant difference between the two control infusions (W=10.5, p=0.561). CS-directed responses showed a significant rise above baseline (one-sample tests against 0; Saline 1: t-test, t=3.97, p=0.042; Saline 2: one-sample Wilcoxon test for non-parametric data, W=16, p=0.042). Two-way linear mixed effects model analyses (session order x recovery phase) on the recovery of HR revealed a significant effect of phase ($F_{(2.6)}$=9.84, p=0.013), with no effect of session order ($F_{(1,3)}$=7.00, p=0.077) and no session order by phase interaction ($F_{(2,6)}$=3.26, p=0.110). Post hoc pairwise comparisons revealed significant differences between Rec20 and Rec40 (p=0.270) and between Rec20 and Rec60 (p=0.240). A: bars represent the mean, with error bars representing the standard error of the mean. Individual data points are presented for each subject, in reference to Table 1 (n=4 for all). B: each point represents the mean (n=4 for all). Significance symbols: * p<0.05, one-sample tests against 0 (as above).

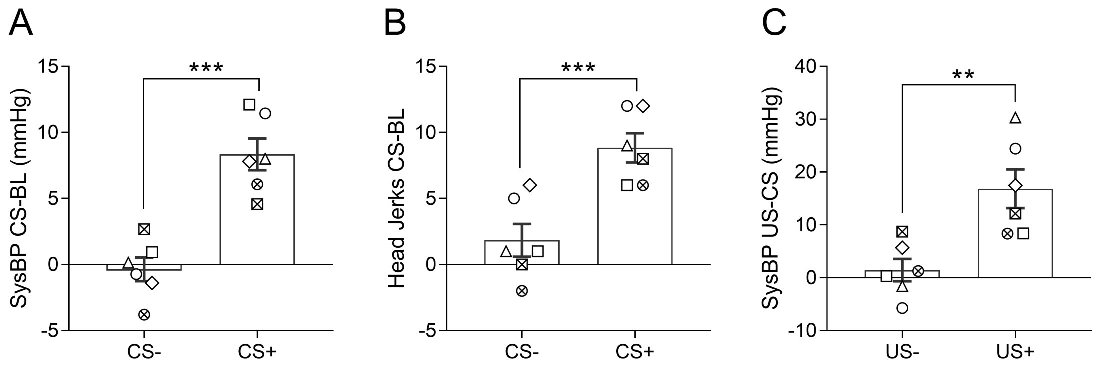

Supplementary Figure 4 – Marmosets show evidence of successful discriminative conditioning to reward. Data presented is the mean of responses of each subject over the 3 discriminative conditioning sessions immediately before drug treatment began. This is presented for CS-directed cardiovascular (sysBP; A), and behavioural responses (B) for both CS^- and CS^+. The US^+-directed cardiovascular response is also presented (sysBP; C), demonstrating a sustained increase in sysBP during marshmallow consumption. Successful discrimination between CS^+ and CS^- was determined using paired t-tests (sysBP: $t=10.2$, $p<0.001$; behaviour: $t=11.91$, $p<0.001$). The same test conducted on cardiovascular responses to the US relative to the preceding CS revealed a significant difference between the US^+ and the US^- ($t=4.33$, $p=0.008$). The bars represent the mean, with the error bars representing the standard error of the mean. Individual data points are presented for each animal as designated in Table 1 (n=6 for all). Significance symbols: ** $p<0.01$, *** $p<0.001$, paired t-tests (as above).

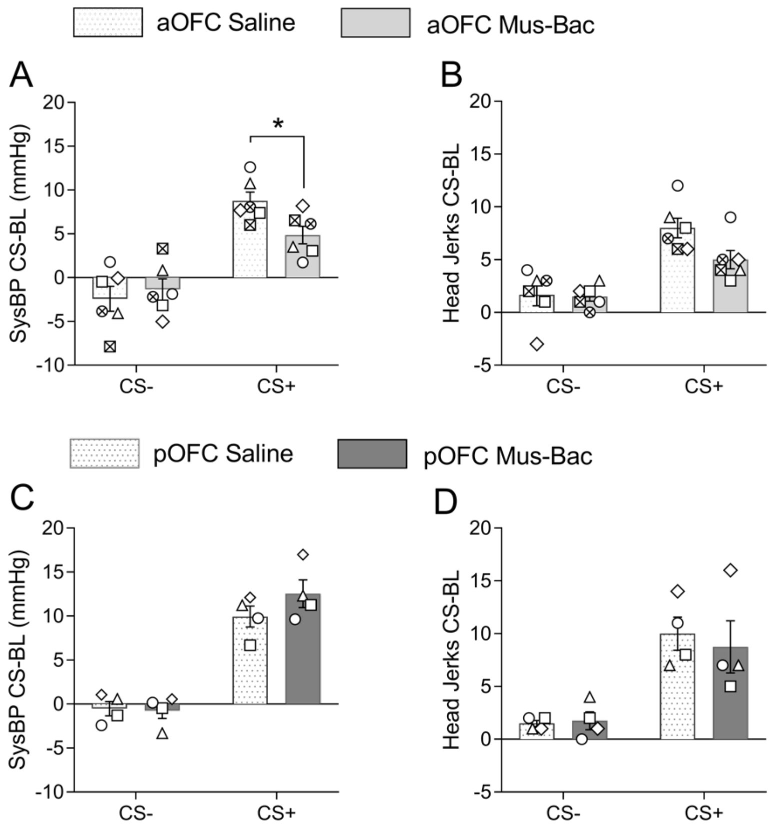

Supplementary Figure 5 – Effects of aOFC and pOFC inactivation on CS-directed responses in the discriminative appetitive Pavlovian conditioning test. Effects of inactivation (Mus-Bac) are compared to the control (Saline) for both aOFC (top, light grey) and pOFC (bottom, dark grey) individually for the CS^- and CS^+ trials. The effects are compared on CS-directed sysBP arousal (A, D) and behaviour (appetitive head jerks; C,F). A three-way linear mixed-effects model analysis (area x treatment x CS-type) revealed a significant area by treatment by CS type interaction for sysBP ($F_{(1,28.42)}$=4.95, p=0.034), a significant main effect of area ($F_{(1,31.88)}$=10.94, p=0.002) and CS ($F_{(1,28.42)}$=137.66, p<0.001), with no further treatment related effects (treatment: F<1; area x treatment: $F_{(1,28.42)}$=2.18, p=0.151; treatment x CS type: F<1). Post hoc pairwise comparisons revealed a significant effect of aOFC inactivation compared to saline on the CS^+ response (p=0.019) but no effect on the CS^- (p=0.499). There were no effects of pOFC inactivation (saline vs mus-bac, CS^+: p=0.186; CS^-: p=0.900). The analysis on behavioural data (head jerks) revealed a significant effect of CS type ($F_{(1,28.28)}$=60.87, p<0.001) but no treatment effects (overall treatment: $F_{(1,28.28)}$=1.81, p=0.189; treatment x area: F<1; treatment x CS type: $F_{(1,28.28)}$=1.95, p=0.173; treatment x area x CS type: F<1). The bars represent the mean, with error bars representing the standard error of the mean. Individual data points are presented for each subject, in reference to Table 1 (n=6 for aOFC, n=4 for pOFC). Significance symbols: * p<0.05, pairwise comparisons (treatment), post hoc for linear mixed effects model analysis (as above).

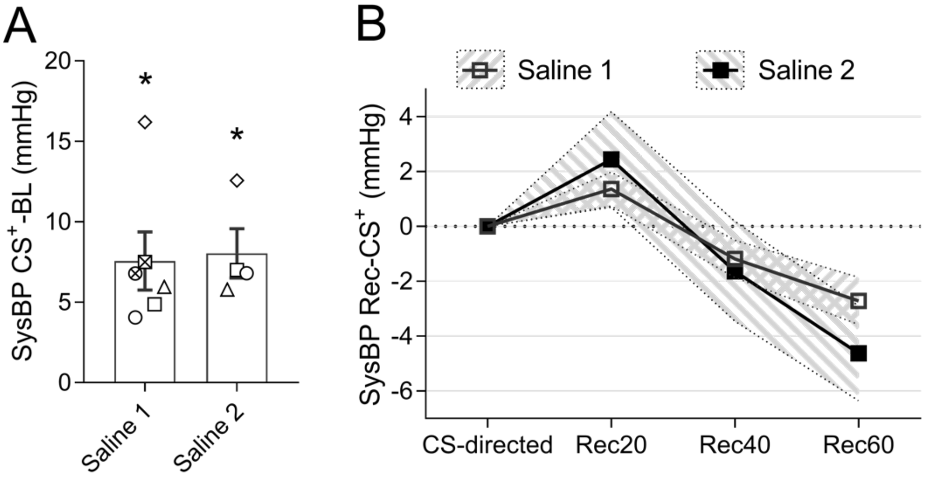

Supplementary Figure 6 – Repeated CS+ presentations with US+ omission do not significantly affect CS-directed responses and are followed by significant recovery in arousal. A. presents the CS+-directed cardiovascular responses in terms of sysBP. The responses are presented for the two saline control sessions (aOFC and pOFC), by order in which they took place rather than by target brain area (there was an inactivation session in-between the two saline sessions). One-sample two-tailed t-tests comparing CS+-directed sysBP against 0 revealed that the increase was significant for both sessions (Saline 1: p=0.017; Saline 2: p=0.014, with Sidak correction for multiple tests). Bars represent the mean, with error bars presenting the standard error. Individual data points for each subject are included. B. presents the recovery of cardiovascular arousal, which is presented as the sysBP relative to the CS-directed response and split over three 20-second bins (Rec20, Rec40 and Rec60; n=6 for saline 1 and n=4 for saline 2). The CS-directed response is presented for reference and was not included in the analysis of recovery. A two-way linear mixed-effects model analysis (recovery phase x session) on sysBP demonstrated a significant effect of recovery phase (phase: $F_{(2.19.33)}$=28.01 p<0.001; session: F<1; session x phase: $F_{(2,19.33)}$=2.00, p=0.163), with post hoc pairwise comparisons revealing significant differences between the early and late phases in recovery (Rec20 vs Rec40: p=0.001; Rec20 vs Rec60: p<0001; Rec 40 vs Rec60: p=0.021). Significance symbols: * p<0.05, one-sample t-tests against 0, as above.
